# The Effectiveness of Reminiscence Therapy on Alleviating Depressive Symptoms in Older Adults: A Systematic Review

**DOI:** 10.3389/fpsyg.2021.709853

**Published:** 2021-08-17

**Authors:** Zhuo Liu, Fan Yang, Yifan Lou, Wei Zhou, Feng Tong

**Affiliations:** ^1^Research Institute of Social Development, Southwestern University of Finance and Economics, Chengdu, China; ^2^School of Social Work, Columbia University, New York, NY, United States; ^3^Party School of Chongqing Municipal Committee of CPC, ChongQing, China; ^4^School of International Law and Sociology, Sichuan International Studies University, Chongqing, China

**Keywords:** older adults, depression symptoms, systematic review, reminiscence therapy, the effectiveness

## Abstract

**Objective:** Depression is one of the most common problems faced by older adults. Reminiscence therapy, defined as using the recall of past events, feelings, and thoughts facilitating pleasure, is one type of psychotherapy that could alleviate depressive feelings among older adults, improve their quality of life, and help them live independently. Reminiscence therapy originated from geriatric psychiatry, and is an effective non-pharmacological intervention that could be structured or unstructured and be conducted individually or in a group. The current systematic review was designed to summarize and review existing evidence on the effect of reminiscence therapy on depression in older adults.

**Methods:** We conducted a systematic review from January 2000 to Mar 2021 using 10 electronic databases in English and Chinese languages, including Medline, Embase, Cinahl, PsychInfo, Cochrane, Web of Science, Google Scholar, Science Direct, CNKI, and WANFANG. We excluded studies that didn't use randomized controlled trials (RCT) from the meta-analysis. The selected studies were scored using the Cochrane Risk of Bias tool. The RevMan 5.0 was used in subgroup analysis depending on how the interventions were classified.

**Results:** We extracted 527 studies based on keyword searches, of which 10 RCTs met inclusion criteria were included in the meta-analysis. The meta-analysis yielded high heterogeneity, and the analyses of significant subgroups showed that reminiscence therapy has a significant effect on relieving depressive symptoms in older adults. Reminiscence therapy benefits older adults with chronic illness and those on antidepressants as well. The effect and cost-effectiveness of group reminiscence therapy were higher than individual reminiscence therapy. And some specific types of group reminiscence therapy have a significant effect on improving depression and secondary outcomes, including life satisfaction. Although the effectiveness of structured and unstructured group reminiscence on depression has no significant differences according to current evidence, the structured therapy is more replicable, generalizable, and user-friendly due to its detailed protocol for new therapists. Furthermore, reminiscence therapy is more effective for older women and older adults with more severe depressive symptoms.

**Conclusion:** Reminiscence therapy significantly increased older adults' remission from depression and quality of life immediately after the intervention. However, the evidence-based protocol and implementation of reminiscence interventions need to be further developed and standardized to facilitate global use. Moreover, it remains unclear on the long-term effect of reminiscence therapy. Based on the limitations of the current study, more rigorous evidence is needed from studies with large sample sizes, RCT design, and longer follow-up periods. Future studies could also explore the effect of different types of reminiscence therapy. Furthermore, qualitative data should be included to better understand older adults' narrative and experiences with reminiscence therapy. Future studies could also investigate the impact of reminiscence therapy on older relatives as a part of outcome measure to explore the efficacious mechanism of reminiscence therapy in alleviating older adults' depressive symptoms.

## Introduction

By 2050, one in six people in the world will be over age 65 (16%), a significant increase from the proportion (9%) of older adults in 2019 (UN, 2019a). The needs of mental health problems of older adults are expected to increase dramatically due to the higher prevalence of older adults in the society (UN, 2019b). Addressing older adults' needs on mental health would benefit older adults and their family members physically, psychologically, and financially.

Depressive symptoms, also called depressive mood, are one of the most common mental health problems in older adults and usually consist of dismay, discomfort, insensitivity, and pessimism. Depressive symptoms in older adults are negative emotional responses to external and internal environmental stimuli, accompanied by signals of reduced mental energy, low spirit, sadness, and misery, which disturb their daily life. The depressive mood is a normal emotional response when people encounter difficulties, but depressive symptoms develop into depression when maintained at a high level over a long period. In severe cases, self-harm and suicide may occur due to depression (McGirr et al., 2007). Depressive symptoms are a potential risk factor for depression in older adults, inducing other severe illnesses and even suicide (Wang and Zhang, 2019). Onder et al. (2005) conducted a cross-sectional survey of 3,976 older adults living at home in 11 European countries and demonstrated that 30.8% of them have depressive symptoms. Previous studies indicate that depression is more fatal to older adults—older adults age 70 and older have the highest suicide rates across all age groups that are closely related to depression (World Health Organization, 2014; Kok and Reynolds, 2017).

Currently, both pharmacological and non-pharmacological treatments are available to treat depression in older adults. A large number of studies have shown that pharmacological treatment has many side effects (Wang et al., 2011; Cotelli et al., 2012; Gil et al., 2019), such as gaining weights, risk for heart disease, etc. Psychosocial methods, on the other hand, are often used by social workers as a key intervention strategy to address older adults' depression symptoms due to its standardized operating procedures, minimum harm and side effects, and cost-effectiveness (Lin et al., 2003; Bohlmeijer et al., 2007; Gil et al., 2019). Life review is both a phenomenon and a method of treatment for old adults with depression, and its impact have interested a growing number of scientists and clinicians (Fry, 1983; Watt and Cappeliez, 2000). It was introduced by Butler in 1963 based on Erikson's psychosocial development theory and Aichley's continuity theory (Butler, 1963). It refers to a treatment process in which older adults are guided to review, re-experience, and generate new interpretations of their past lives in order to help them better understand themselves, increase self-esteem, and promote socialization (Hsieh and Wang, 2003). Reminiscence therapy can be administered by either individual or group reminiscence therapy based on the needs of participants. Moreover, Westerhof et al. (2010) divided reminiscence therapy into simple reminiscence, life review, and life review therapy, according to the depth of participants' memories. Simple reminiscence therapy is suitable for older adults with psychological problems who do not need pharmacological treatment. In contrast, life review requires an implementer to design an intervention plan, ask questions, formulate processes and set goals based on older adults' circumstances. Life-review therapy is a highly structured psychotherapy method suitable for older adults suffering from depression and other mental illnesses (Fan and Li, 2014).

Although a large number of researches exploring the effectiveness of reminiscence therapy on depression in older adults, the efficacy of reminiscence therapy as a psychological intervention is still inconclusive. Although some studies found that non-pharmacological interventions are beneficial in relieving depression symptoms (i.e., Duru-Aşiret and Kapucu, 2016), other studies found no evidence in terms of the efficacy of reminiscence therapy for depression (Wang et al., 2007). We acknowledge that there are two last systematic review on this topic in 2019 (Gil et al., 2019; Sahu et al., 2019), and we integrated them into this review dates. The two reviews both found that reminiscence therapy has potential efficacy for maintaining cognition and decreasing depressive symptoms in the target population. Although the two studies synthesized randomized clinical trials and quasi-experimental studies, they did not perform meta-analyses and thus their results might be biased and have limited generalizability. Further, Chinese scholars have reported in a literature review that eight studies showed reminiscence therapy has no significant effect on older adults' depression (Chen et al., 2012). However, their judgment of reminiscence therapy's efficacy might have been affected by differences in the application of reminiscence therapy in China due to biases caused by differences in older adults based on regions and customs because of several ethnic integration attempts. Moreover, the theory and localization of intervention programs are yet to be accomplished in China (Wu and Hu, 2017).

Because of the above limitations, this study included evidence from Chinese and English randomized clinical trials (RCT) on reminiscence therapy interventions for older adults' depressive symptoms conducted between January 2000 and April 2021. With the Preferred Reporting Items for Systematic Reviews and Meta-Analyses (PRISMA) 2020 (Page et al., 2021), this study was designed to explore the overall effect of reminiscence therapy on depressive symptoms in older adults and present the latest, highly valid evidence for reminiscence therapy's efficacy in alleviating depressive symptoms of older adults.

## Methods

### Eligibility Criteria

This study was registered in CAMPBELL CHINA. The study's inclusion criteria were decided according to the principles of “PICOS” in the Cochrane System Evaluation Manual (Higgins, 2011) as described below. (1) The participants in this study were older adults over 50 years of age. In the included literature, all participants were over 60 years of age, except for one study in which the subjects were 51–90 years of age (but the mean age was 64.3 years). The reasons for using 50 years as the criterion for older adults are: (i) the inclusion criteria of Pot extend the definition of “older adults” to those over 50 years of age due to the limitations of the recruitment method. (ii) In China, the retirement age for female workers is 50 years old, and retirement is generally considered to be a step into old age. (2) The purpose of the intervention is to alleviate depressive symptoms in older adults. (3) There are standardized and credible research goals and reported conclusion data of the intervention effect on depression symptoms. (4) The intervention used is reminiscence therapy. (5) The depressive symptoms are mild to moderate. (6) Randomized Controlled Trial (RCT). (7) The language of the article is Chinese or English.

The exclusion criteria were: (1) studies published in languages other than Chinese or English, (2) studies on older adults suffering from severe depressive symptoms or are seriously ill, (3) studies that do not report both pre- and post-tests on depressive symptoms; (4) studies with a sample size of lower than 20 participants, and (5) studies with patients with neurological diseases or cognitive impairments.

### Search Strategy

We searched Medline, Embase, Cinahl, PsychInfo, Cochrane, Web of Science, Google Scholar, Science Direct, CNKI, and WANFANG databases. We searched for Chinese and English language papers published between January 2000 to March 2021. We searched all the databases using the keywords “reminiscence therapy” AND “elderly depression emotion/senile depression symptoms” and other related synonyms.

### Selection Process

First, three researchers independently searched the databases using the selected keywords. Then, two researchers read the titles of the extracted studies for initial screening. Following this, three researchers read the abstracts of the identified studies and excluded studies that were inconsistent with the inclusion criteria. After that, three researchers read the full text of the included literature and extracted valid data from the article, and inconsistent literature was again excluded from the study. Finally, one researcher summarized and verified the final data.

### Risk of Bias Assessment

The trial quality was screened by two researchers, and the risk of bias was determined based on Cochrane's risk of bias tool. The evaluation principles of Cochrane's risk of bias tool involve six perspectives: selection bias, performance bias, measurement bias, data bias, reporting bias, and other biases (Higgins et al., 2011). Accordingly, Cochrane's risk of bias for this study's data included whether the choice was made in random sequence, whether the allocation of groups was concealed, whether the implementation and measurement were blind, whether the data was complete, whether the analysis was comprehensive, and whether there were other biases. The included articles were evaluated from these six perspectives to ensure the overall validity of the evidence.

These researchers analyzed the data to obtain a bias analysis and quality evaluation report according to the PRISMA2020 statement standard using the RevMan 5.0 (Review Manager) software provided by the Cochrane Collaboration.

## Results

### Study Selection

**Figure 1:**
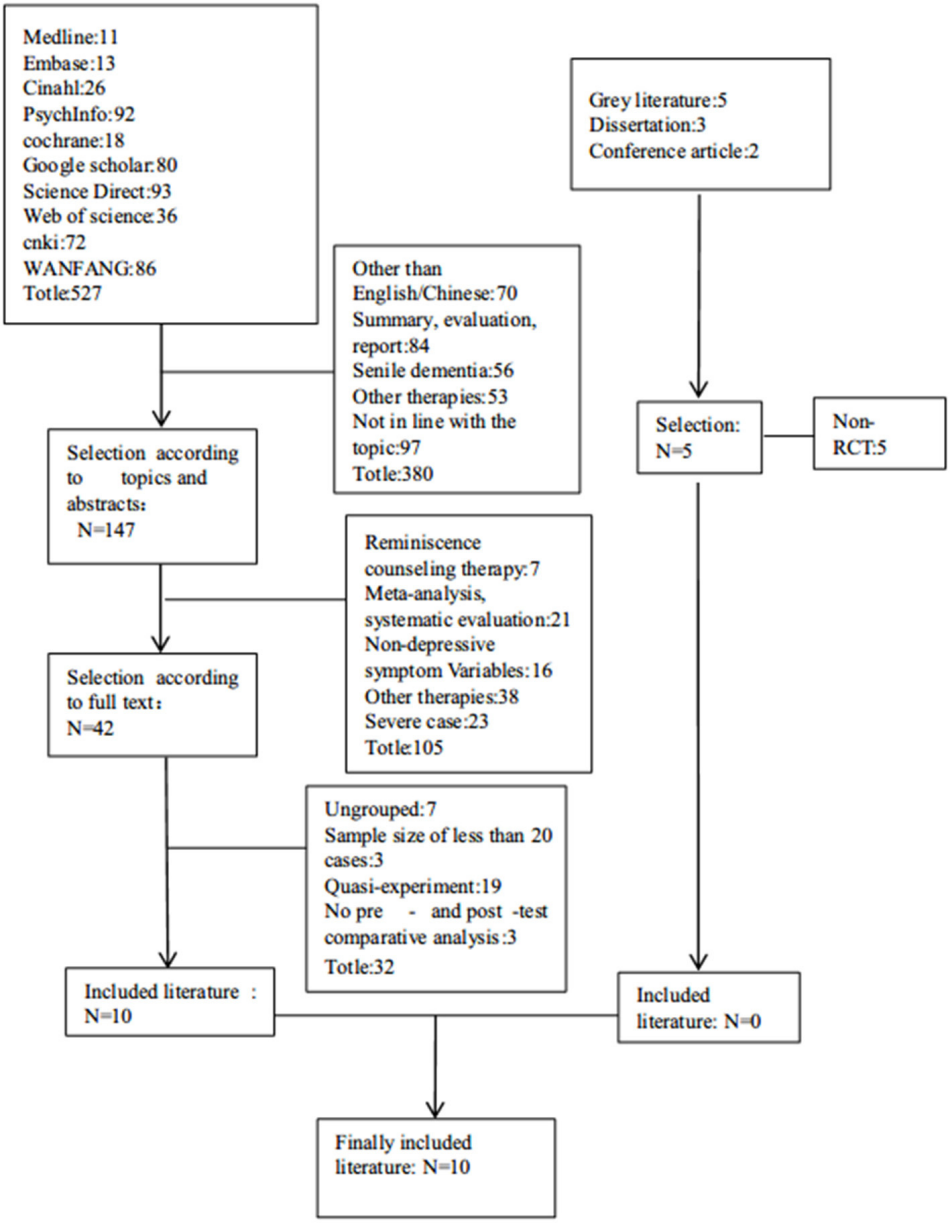
Flow chart of the literature selection of reminiscence therapy intervention for elderly depression.

A total of 527 related articles were identified and retrieved based on the above methods (see Figure 1). Ten studies met the inclusion criteria and were included in the review. In the included studies, 453 older adults aged over 50 years received an intervention. Nine studies of the ten studies listed the content of reminiscence therapy, and three had a sample size >100 (Pot et al., 2010; Zhou et al., 2012; Viguer et al., 2017). Figure 1 provides a flow chart of literature selection on reminiscence therapy interventions for older adults with depressive symptoms.

### Study Characteristics

Of the studies included in the meta-analysis, four were identified from the Chinese literature (Wang et al., 2011, 2020; Yue and Chen, 2013; Cai, 2018). The geographical distribution of studies included six conducted in China, one in the Netherlands (Pot et al., 2010), one in the Dominican Republic (Viguer et al., 2017), one in Iran (Hamzehzadeh et al., 2018), and one in Malaysia (Syed-Elias et al., 2020). The research sites included three hospital studies (Yue and Chen, 2013; Cai, 2018; Wang et al., 2020), three community studies (Wang et al., 2011; Zhou et al., 2012; Syed-Elias et al., 2020) and four institutional studies (Chiang et al., 2010; Pot et al., 2010; Viguer et al., 2017; Hamzehzadeh et al., 2018). The included studies consisted of the group and individual interventions, of which eight studies using group reminiscence and two using one-to-one reminiscence interventions (Wang et al., 2011; Cai, 2018). The intervention targets include older inpatients in hospitals, older adults in nursing homes, empty-nesters in the community, and older adults in the community.

Depressive symptoms were the primary outcome measured in all the studies using different depression scales, used the following tools: The Geriatric Depression Scale (GDS) consists of 30 questions, and each question answer “Yes” and “No.” The Center for Epidemiological Studies Depression Scale(CES-D). It is a 20-item self-report scale developed to measure a person's emotional performance in the past week. Those studies also measured other outcomes including self-report life satisfaction, happiness, self-esteem, and loneliness. Eight studies included information on the persons who conduct the interventions, including nurses, psychological counselors, social workers, postgraduates, and experts that had received training before implementing the intervention.

The shortest intervention period was 3 weeks (Wang et al., 2020). Six studies used pre- and post-evaluation, whereas most studies had a regular intervention period lasting 3–8 weeks. Interventions were conducted once a week or once every 2 weeks. One study clearly stated that the enrolled participants were taking antidepressants (Wang et al., 2020), and two studies indicated that older adult participants had chronic diseases (Cai, 2018; Wang et al., 2020). Four studies had a follow-up of 3–9 months after the intervention (Chiang et al., 2010; Pot et al., 2010; Viguer et al., 2017; Syed-Elias et al., 2020).

### Inclusion in the Study

We classified the characteristics of the included studies based on the PICOS model (Table 1). In all the studies, only the intervention groups were provided reminiscence therapy. The control group received routine care, health education, and conducted general activities and community services.

**Table 1:** The characteristics of the included studies based on the PICOSS model.

**References**	**Participants (excluding the number of people lost to follow-up)**	**Intervention measure**	**Control measure**	**Outcome**	**Evidence environment**	**Instructor**	**Intervention technique**	**Intervention form**
Wang et al. (2011)	Hefei, China, 82 empty-nesters in the community Age ≥ 60 42 participants in the intervention group, with 59.5% of women; 40 participants in the control group, with 55% of women No loss to follow-up rate.	Individual reminiscence therapy based on routine community care Once a week in a 45–60-min session, 8 weeks; specify the reminiscence topic.	Routine community care; community nurse visits home once a month to resolve existing health problems	GDS MUNSH (Memorial University of Newfoundland Scale of Happiness)	Older adults in community; home	Trained community nurses with professional titles above nurse practitioner	The intervention staff meet once every 2 weeks to ensure the standardization of interventions and life review; test before and after 8 weeks.	Individual reminiscence therapy
Yue and Chen (2013)	Hangzhou, China; 80 patients with mild to moderate depressive symptoms; 62–80 years old, with an average age of 71.2 ± 15.4 40 participants in the intervention group, with 50% of women; 40 participants in the control group, with 55% of women No loss to follow-up rate.	Group reminiscence therapy based on routine care Once a week in a 40–50-min session, 6 weeks Specify the reminiscence topic.	Provide routine care and random health education	GDS LSI-A	Hospital	Trained, patient and responsible nursing staff	Combine with the actual situation to design a structured group reminiscence therapy; test before and after 6 weeks	Structured group reminiscence therapy
Cai (2018)	Changsha, China, 61 elderly patients with chronic diseases Age ≥ 60 31 participants in the intervention group and 30 participants in the control group.	Individual reminiscence therapy based on routine care Once a week in a 30–45-min session, 6 weeks Specify the reminiscence topic.	Routine care; taking drugs as prescribed by doctors; life, diet and psychological care; health education	GDS UCLA (Loneliness Scale, University of California at Los Angels)	The patient interview room in the hospital ward	1 trained head nurse and 5 charge nurses; specify the precautions	Set up a reminiscence topic on the basis of consulting patients and their family members with reference to relevant literature; life review; test before and after 6 weeks.	Individual reminiscence therapy
Wang et al. (2020)	Yancheng, Jiangsu Province, China age ≥ 60 30 participants in the intervention group (11 males and 19 females) and 28 participants in the control group (10 males and 18 females).	Group reminiscence therapy based on routine care; Twice a week in a 40–60-min session with 6–9 people, 6 weeks Specify the reminiscence topic.	Routine nursing care for depression based on taking antidepressants	GDS, LSI-A	Hospital	2 systematically trained psychiatric clinical nurses specialists take turns to preside over the intervention	Determine the reminiscence topic by referring to relevant researches on reminiscence therapy at home and abroad, combined by the results of interviews with patients; test around 3 weeks	Group reminiscence therapy
Chiang et al. (2010)	Taipei, Taiwan Province, China Age ≥ 65 45 participants in the intervention group and 47 participants in the control group.	Once a week in a 90-min session, 8 weeks; specify the reminiscence topic.	Waiting list control	CES-D, SCL-90-R RULS-V3 MMSE	Nursing home the recreation room	A master's prepared student, A co-leader training consisted of 54 h of didactic training followed by the reminiscence group therapy manual.	3 months follow up	Group reminiscence therapy
Pot et al. (2010)	Netherlands Age > 50, with an average age of 64 83 participants in the intervention group, with a female ratio of 73.5%; 88 participants in the control group, with a female ratio of 71.6%.	Each session is centered on a topic related to the course of life, 12 sessions of 2 h each; specify the reminiscence topic.	20-min educational video supplied information about factors and skills	CES-D, HADS, MANSA, Mastery Scale RFS	Institute of mental health and addiction	Two mental health care professionals with a therapeutic background or a qualification in behavioral sciences or social work, a 2-day training program in advance and 1 day's training during the course	3 or 9 months follow up	Group life review prevention
Zhou et al. (2012)	Changsha, China Age > 65 59 participants in the intervention group (25 males and 41 females) and 66 participants in the control group (25 males and 34 females).	Group reminiscence therapy based on health education; once a week in a 90–120-min session, 6 weeks; weekly topics for group reminiscence therapy; specify the reminiscence topic.	Three health education Sessions: one every 2 weeks lasting 30–45 min each	GDS, The Chinese version of SES, The Chinese version of ABS, Analytic strategy	Community health service centers or neighborhoods	Trained community nurses	Group psychological therapy techniques; test before and after 6 weeks.	Group reminiscence therapy
Viguer et al. (2017)	The Dominican Republic Age ≥ 65 80 participants in the intervention group, with a female ratio of 56.3%; 80 participants in the control group, with a female ratio of 51.2%.	A 10-session intervention, the procedure was standardized with guidelines that included exercises and structured input and consisted of 10 weekly group sessions lasting 2 h each; specify the reminiscence topic.	Complete the same measures at the same time as the intervention group, but they will be not exposed to the training	GDS-30 LSI-A PWBS	Healthcare centers	A trained psychologist	Standardized with guidelines that included exercises and structured input; 3 months follow up	Group sessions
Hamzehzadeh et al. (2018)	Tearan, Iran Age ≥ 60 Female, 10 participants in the intervention group and 11 participants in the control group; no loss to follow-up rate.	8 sessions, 1 h per session and each week 2 sessions, 4 weeks in total; fail to specify the reminiscence topic.	Greeting and regular care	MMSE, GDS, LOT	Nursing home	Examiner	Use 8 intervention sessions focused on different topics and purposes; test before and after 1 month.	Group reminiscence therapy
Syed-Elias et al. (2020)	Malaysia Age ≥ 60 18 participants in the intervention group, with a female ratio of 50%; 16 participants in the control group, with a female ratio of 56%.	Once a week in a 60–90-min session, 6 weeks; specify the reminiscence topic	Attention control activities	UCLA, GAS, M-GDS-14	RACF	The facilitator who led the SRT session	The researcher has developed the detailed manual each week; finding the meaning of life; 3 months follow up	Group reminiscence therapy

### Methodological Validity

**Figure 2:**
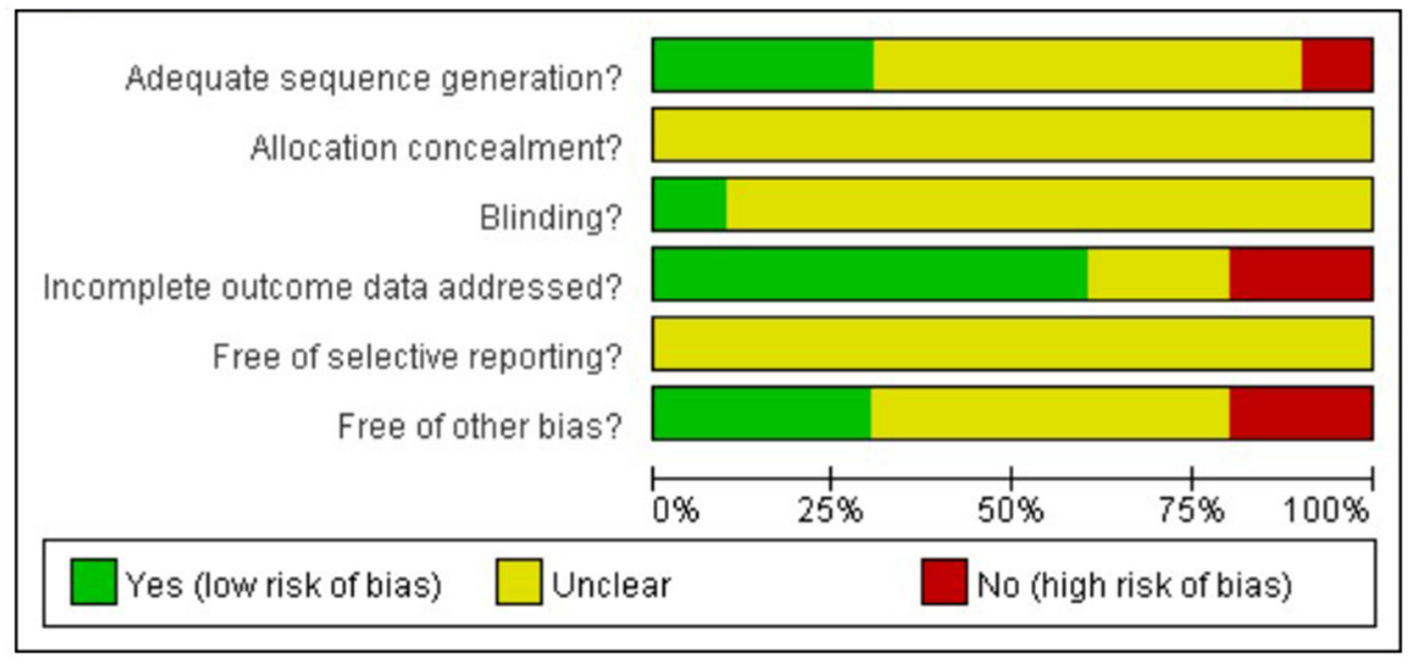
Risk of bias diagram of included studies.

In terms of the risk of bias in the included studies (Figure 2), four studies notified the random sequences. None of the 10 articles specified the allocation concealment, nor did they notify whether the studies were blind or not. The selective reporting of the evidence was not clearly stated. Moreover, three studies were low in other risks of bias (Chiang et al., 2010; Viguer et al., 2017; Syed-Elias et al., 2020). Overall, the quality of the 10 included articles was acceptable.

### Results of Syntheses

#### Comparison of Older Adults' Depression Symptoms

Four studies maintained high data integrity(no data loss) (Wang et al., 2011; Yue and Chen, 2013; Hamzehzadeh et al., 2018; Syed-Elias et al., 2020). One study reported 11.1% data loss after completing the test and 15.8% data loss during the follow-up period (Pot et al., 2010), and five articles reported the accurate lost data rate. 3.1% date loss (Zhou et al.), 10.29% date loss (Cai, 2018), 4.76% date loss (Viguer et al., 2017); 6.45% date loss (Wang et al., 2020) and 29.23% date loss (Chiang et al., 2010). However, the study's results showed that reminiscence therapy is effective for older adults suffering from depression and was unaffected by the data loss. Nine of the included studies used the Geriatric Depression Scale (GDS), whereas another study used the Center for Epidemiologic Studies Depression Scale (CES-D; Pot et al., 2010) for assessing depression. All the participants of one study were men (Chiang et al., 2010), and those of another study were all women (Hamzehzadeh et al., 2018).

**Figure 3:**
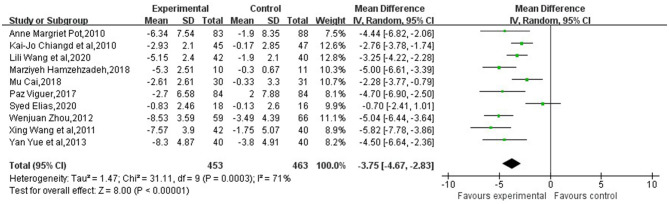
Forest plot of the overall effect of reminiscence therapy on relieving depressive symptoms.

**Figure 4:**
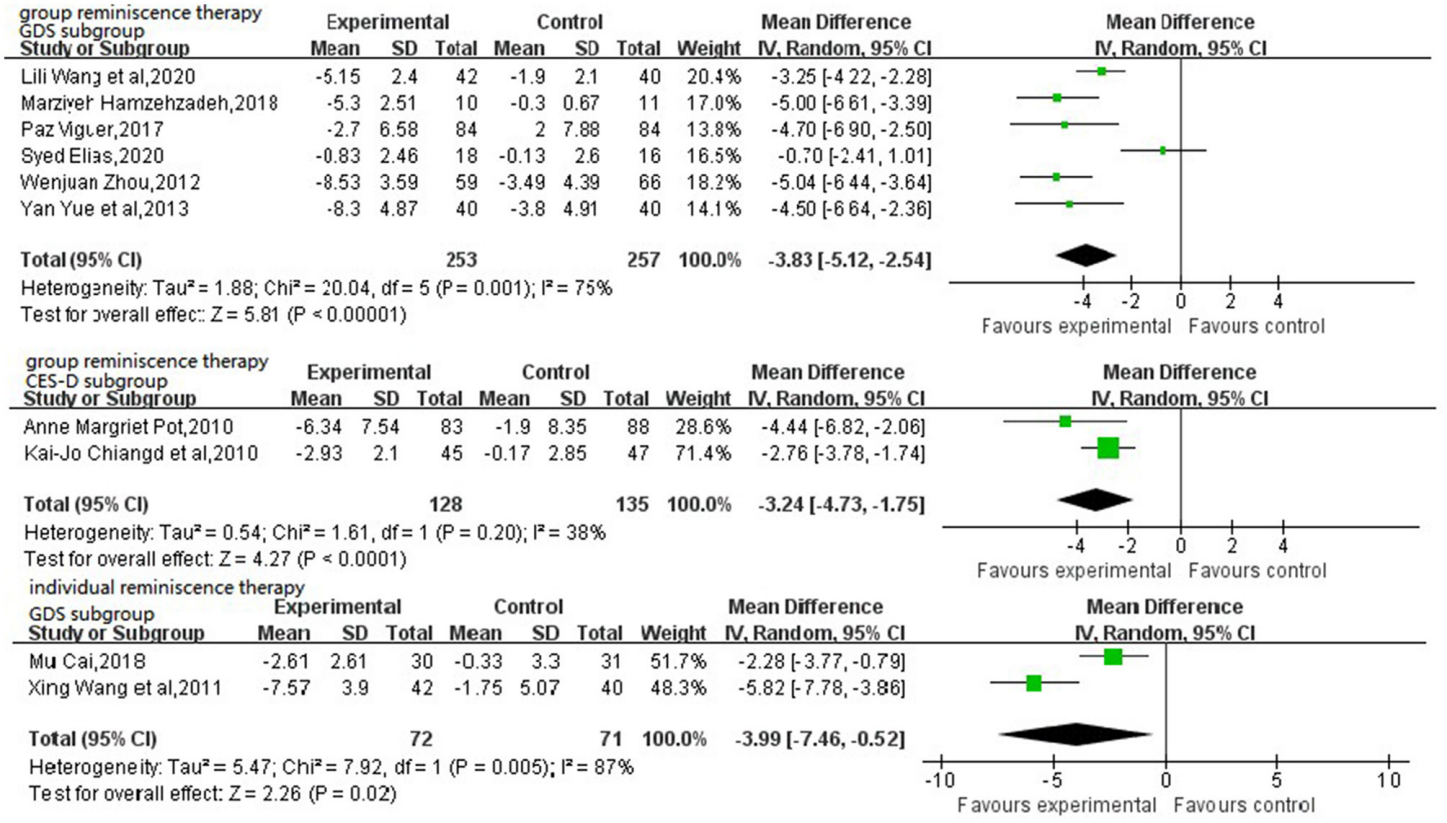
Forest plot of the effect of group and individual reminiscence therapy on relieving depression symptoms.

The forest plots (Figure 3) synthesized the overall effects both group and individual reminiscence, which indicated that reminiscence therapy has a significant effect on relieving depressive symptoms in older adults [MD = −3.75, 95% CI (−4.67, −2.83)]. Although random effect model has been conducted, it still indicated *P* < 0.001 and *I*^2^ ≥ 50%, with clinical heterogeneity. For reducing heterogeneity, random effect model and subgroup analysis by same scale have been implemented. However, group and individual intervention scores indicated that both types of therapies were effective in reducing depressive symptoms [MD = −3.83, 95% CI (−5.12, −2.54) (group reminiscence therapy GDS subgroup)], [MD = −3.24, 95% CI (−4.73, −1.75) (Group reminiscence therapy CES-D subgroup)]and [MD = −3.99, 95% CI (−7.46, −0.52) (Individual reminiscence therapy GDS subgroup)] (Figure 4). But there was still clinical heterogeneity from the perspective of risk ratio (group *I*^2^ =75, ≥50%). which mainly comes from the methodological heterogeneity, while the heterogeneity of individual intervention is 87%, which is due to the heterogeneity of the study subjects, and the base-line of these two individual groups is quite different. one study targeting elderly living alone was 17.90 ± 4.42 and 17.07 ± 3.52 (Cai, 2018), Another study targeting elderly chronically ill patients was 8.95 ± 2.81 and 8.61 ± 3.13 (Wang et al., 2011). Overall, these findings indicated that reminiscence therapy significantly affected relieving depression symptoms in older adults in general.

#### Comparison of Life Satisfaction Index

**Figure 5:**
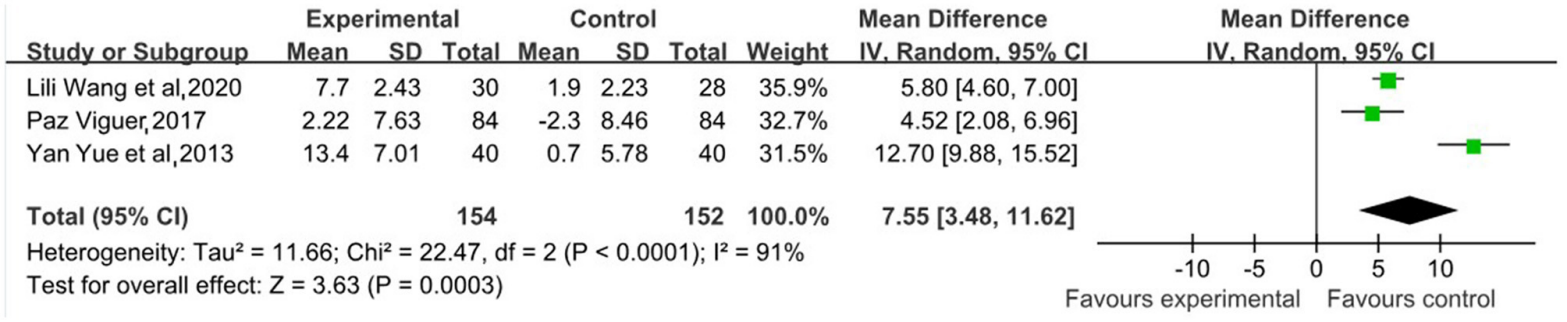
Forest plot of the overall effect of reminiscence therapy on improving life satisfaction.

The meta-analysis results shown in Figure 5 indicate that reminiscence therapy significantly improved older adult's life satisfaction at a p-level of 0.001 [MD = 7.55, 95%CI (3.48, 11.62)]. But there was still clinical heterogeneity (*I*^2^ = 91, ≥50%), which mainly comes from heterogeneity of subjects, one study subjects (Yue and Chen, 2013) is different from other two. The 95% CI of the results of the included 3 studies were distributed on right sides of the axis, suggesting significant positive correlation between the overall effect of the interventions, and all of them had significant effects.

## Discussion

We systematically evaluated 10 reminiscence therapy intervention trials. The results are consistent with previous studies concluding that the depressive symptoms of older adults were significantly relieved after the intervention using reminiscence therapy (Hsieh and Wang, 2003; Chen et al., 2012; He, 2018). As a special branch of reminiscence therapy, the effectiveness of Spiritual Reminiscence Therapy(SRT) for depression is still inconclusive, but the within-group's results showed a significant reduction in M-GDS-14 scores from pre-test to 3-month follow-up for both groups. This means that the SRT program may have some positive effect to attenuate depression (Syed-Elias et al., 2020), and the results of meta-analysis showed that the effect size was the smallest [MD = −0.70, 95% CI (−2.41, 1.01)].

The overall effect and cost-effectiveness of group reminiscence therapy are higher than individual reminiscence therapy. Among the 10 studies, only two used individual reminiscence therapy (Wang et al., 2011; Cai, 2018). Comparing the intervention effects of group and individual reminiscence therapies, individual reminiscence therapy's effect size [MD = −3.99] was smaller than that of group therapy [MD = −3.83]. There are several factors may affect the intervention effect of an individualized approach (Bai and Shen, 2017). First, although individual reminiscence therapy can be more tailored to meet the individual needs, it is more time-consuming and labor-intensive compared to group therapy. Second, individual reminiscence therapy cannot provide an interactive platform for older adults. On the other hand, group reminiscence therapy focuses more on facilitating the interactions between older adults and environments and helping build the social networks and acquire a sense of self-identity and belonging (Housden, 2009). Moreover, we found that group reminiscence therapy is effective for older adults with mild and moderate depression, whereas individual reminiscence therapy is only effective for older adults with mild depression (Bai and Shen, 2017). Also, group reminiscence therapy is more cost-effective than individual therapy due to its lower financial input per individual (Burnside and Haight, 1994). However, it should be noted that the direct comparing between group and individual reminiscence therapies lacks appropriate methodological support.

Among the eight studies that adopted group reminiscence therapy, one employed structured group reminiscence therapy (Yue and Chen, 2013), in which the implementers made adequate plans and preparations in advance, tailored the activities according to the needs of the group, and facilitated active participations of the older adults (Yue and Chen, 2013). Fry (1983) argued that structured group reminiscence therapy is significantly better than non-structured one because structured group reminiscence therapy requires older adults to organize and integrate their spontaneous and evoked memories within a cognitive framework. The framework is informative to older adults and responds to their communication needs in a very supportive social therapy context. In addition, the structured therapy is more replicable, generalizable, and user-friendly due to its detailed protocol for new therapists (Yue and Chen, 2013). Moreover, studies have shown that women and older adults with relatively more severe depression demonstrated better treatment effects, perhaps because women are more likely to accept and participate in reminiscence therapy (Pot et al., 2010). However, there is very limited studies on structured therapy that could be a direction of future research.

Six of the included studies had intervention durations of 6–8 weeks (Chiang et al., 2010; Wang et al., 2011; Zhou et al., 2012; Yue and Chen, 2013; Cai, 2018; Syed-Elias et al., 2020). The four studies conducted in Changsha and Taipei of China, Netherlands, and the Dominican Republic had over 90 min of intervention per session (Chiang et al., 2010; Pot et al., 2010; Zhou et al., 2012; Hamzehzadeh et al., 2018), whereas the four studies conducted in Hefei, Hangzhou, Changsha, and Jiangsu in China had an intervention time <60 min per session (Wang et al., 2011, 2020; Yue and Chen, 2013; Cai, 2018). There was no evidence for a significant relationship between a single intervention's duration and the intervention effect. However, it should be noted that the physical health of older adults might prevent them from participating in activities that last a long period of time. As such, the intervention effects of reminiscence therapy might be lower if the time required for a single intervention is very long.

Furthermore, five studies conducted 1–6 months follow-up to understand how long the intervention effects last. In general, they reported that the effect of reminiscence therapy on relieving depression symptoms persisted (Chiang et al., 2010; Pot et al., 2010; Viguer et al., 2017; Hamzehzadeh et al., 2018; Syed-Elias et al., 2020), but gradually decrease over time (Viguer et al., 2017). Based on these results, this review, similar to the previous reviews, concluded that the effects of optimal treatment time, length and frequency of treatment, and reduction of treatment effects remain unclear (Lin et al., 2003). Moreover, since the tested follow-up period is still relatively shorter than follow-up durations used in other intervention study, the long-term effects of the reminiscence theory remain unclear.

Combining the above graph with the remission of depressive symptoms from reminiscence therapy shows that there is a negative correlation between the elderly's depressive symptoms and their life satisfaction, indicating that the elderly have reduced depressive symptoms and their life satisfaction has increased. However, It is encouraging that reminiscence therapy also had positive impact on older adults' multi-dimensional psychological-well-being, including life satisfaction, loneliness, anxiety, and happiness (Chiang et al., 2010; Wang et al., 2011; Viguer et al., 2017). Three studies used the Life Satisfaction Index A (LSI-A) and reported that the LSI-A score of older adults who received the intervention increased significantly. Moreover, as depression symptoms decreased, the life satisfaction scored improved significantly in the treatment group compared to the control group. Therefore, we concluded that reminiscence therapy has a positive effect on improving the life satisfaction of older adults (Yue and Chen, 2013; Viguer et al., 2017; Wang et al., 2020). Moreover, there are three studies used the UCLA Loneliness Scale and reported that the loneliness in older adults decreased after reminiscence therapy (Chiang et al., 2010; Cai, 2018; Syed-Elias et al., 2020). One of the articles using the UCLA scale showed that the experimental group decreased from 44.53 ± 4.32 to 39.22 ± 4.18 after the intervention, and the control group decreased from 43.67 ± 3.78 to 42.75 ± 3.79 after the intervention, *p* < 0.001 (Cai, 2018). Another one also used UCLA, *p* < 0.01, Effect Size = 0.25 (Syed-Elias et al., 2020). The last one using the RULS-V3 scale, which had a result after completing the test (*z* = −27.26, *p* < 0.0001) and follow-up results (*z* = −22.75, *p* < 0.0001) (Chiang et al., 2010). Additionally, two studies focusing on anxiety using the Hospital Anxiety and Depression Scale (HADS-A) and the Geriatric Anxiety Scale (GAS), respectively, reported that anxiety did not differ significantly between the treatment and control groups (Statistically significant at *p* > 0.01) (Pot et al., 2010; Syed-Elias et al., 2020). There are three research studied happiness as an outcome, using the Memorial University of Newfoundland Scale of Happiness. Reminiscence therapy was effective in enhancing well-being, 21.90 ± 5.65 and 32.69 ± 6.95 before and after the intervention in the experimental group and 21.43 ± 6.06 and 22.80 ± 6.32 before and after the intervention in the control group, *p* < 0.001 (Wang et al., 2011), the Symptoms Checklist-90-R, *z* = −10.63, *p* < 0.0001 (Chiang et al., 2010) and the Psychological Well-Being Scales(mean difference = 0.10, SE = 0.10, *p* = 0.009, *d* = −0.19) (Viguer et al., 2017). Therefore, reminiscence therapy is evident to be an effective intervention that can relieve depression symptoms, increase life satisfaction and psychological well-being, and reduce loneliness of older adults (Hang et al., 2011; Qin et al., 2017).

There are certain variations of the sample characteristics in the included RCTs. One of the studies conducted reminisce therapy with participants taking prescribed antidepressants by physicians (Wang et al., 2020). And there are two studies sampled older adults with chronic illnesses (Cai, 2018; Wang et al., 2020). We included those two studies in final review for multiple reasons. First, appropriate measures were taken during the intervention process in these studies to control for chronic illnesses and pharmacotherapy. Second, those two characteristics are not uncommon among older adults with depression symptoms: chronic illnesses are common and almost inevitable in older adults, and pharmacotherapy remains the primary treatment for older adults' depression and other mental illnesses (Cai, 2018; Wang et al., 2020). Nevertheless, those studies indicated that reminiscence therapy has a significant positive effect on alleviating depressive symptoms even if older adults were suffering from chronic illnesses or were taking antidepressants (Cai, 2018; Wang et al., 2020). However, more evidence from larger-scale studies are needed to support this conclusion. In addition, older relatives as a very important influence factor, There is few studies investigate the impact of reminiscence therapy on older relatives as a part of outcome measure to explore the efficacious mechanism of reminiscence therapy in alleviating older adults' depressive symptoms.

### Limitations and Future Research Directions

It should be noted that this research only included published literature on RCTs in Chinese and English, which might have caused a bias due to incomplete literature collection and specific differences in the included studies related to sample selection, trial design, and validity. Despite this limitation, we systematically reviewed the evidence for the effectiveness of reminiscence therapy and identified several gaps in current evidence, which has both research and practice implications.

There are certain limitations of the included studies. First, it should be noted that it is difficult to compare the effects of interventions of included RCTs due to lack of standardized intervention procedure. For example, there were also differences in intervention methods, and intervention durations, and follow-up period. Also, the studies conducted reminiscence therapy in many different locations, including community centers, medical centers, and care institutions for older adults, resulting in differences in evaluating intervention effects. Second, although the results of this systematic review indicated that the short-term intervention effect of reminiscence therapy was highly significant, reminiscence therapy might merely provide temporary relief to older adults' mental health problems. Therefore, the long-term effect of reminiscence therapy on alleviating depression needs to be investigated by more rigorous research. Third, research results reviewed in this article indicated that the reminiscence therapy implementation leader played a crucial role in the treatment effect (Yue and Chen, 2013). However, only one study involved professional psychologists in guiding treatment (Chiang et al., 2010). Therefore, there is a need to conduct more research on the influence of the leader's ability on treatment outcomes. Forth, the quality of the Chinese research included in this article is not outstanding, as the research on reminiscence therapy and interventions in older adults' depressive symptoms in China is still emerging. Fifth, some of the included studies had a small sample size, and only three studies have a sample size of more than 100. Larger-scale studies are needed in order to have decent statistical power to prove the effectiveness. Last but not least, because of the variety of intervention methods in the included evidence, there may be methodological heterogeneity, and the included scales are not completely uniform, this may also lead to measurement heterogeneity.

Reminiscence therapy, as an evidence-based intervention, may play a more critical role in an aging society for relieving symptoms of depression in older adults with more improvements in the future. First, the duration of a single intervention should be shortened if there is any “oldest old” in the intervention sample. And specific plans should be developed based on the characteristics of older adult population depending on where the treatment is conducted. Second, the reminiscence intervention protocol must be further standardized to promote its application easier for beginners to use. Third, the interventions' implementers should not be limited to nurses. Gerontological social workers and psychologists could also be the persons who conducted the interventions. Third, the intervention implementers should be proficient in professional knowledge and skills of conducting reminiscence therapy and have psychological counseling skills, especially the culturally-competent skills. Implementer who conduct the intervention should have cultural competency in the culture of the participants they serve, so that they can make provide culturally-appropriate responses to older adults' problems during the reminiscence therapy process. Finally, the impact of reminiscence therapy on older relatives should be investigated as a part of outcome measure to explore the efficacious mechanism of reminiscence therapy in alleviating older adults' depressive symptoms.

## Data Availability Statement

The original contributions presented in the study are included in the article/supplementary material, further inquiries can be directed to the corresponding author/s.

## Author Contributions

ZL, FY, FT, and YL drafted the systematic review protocol. FY, FT, and WZ conducted the search, selection of records, and data extraction. ZL, YL, and FT critically revised it for important intellectual content. All authors contributed to manuscript revision, read, and approved the submitted version.

## Conflict of Interest

The authors declare that the research was conducted in the absence of any commercial or financial relationships that could be construed as a potential conflict of interest.

## Publisher's Note

All claims expressed in this article are solely those of the authors and do not necessarily represent those of their affiliated organizations, or those of the publisher, the editors and the reviewers. Any product that may be evaluated in this article, or claim that may be made by its manufacturer, is not guaranteed or endorsed by the publisher.

## References

[B1] Bai Z. F. Shen J. (2017). Progress of research on the application of reminiscence therapy in geriatric depression. J. Nurs. 24, 28–30. 10.16460/j.issn1008-9969.2017.21.028

[B2] Bohlmeijer E. Roemer M. Cuijpers P. Smit F. (2007). The effects of reminiscence on psychological well-being in older adults: a meta-analysis. Aging Mental Health 11, 291–300. 10.1080/13607860600963547 17558580

[B3] Burnside I. Haight B. (1994). Reminiscence and life review: therapeutic interventions for older people. Nurse. Pract. 19, 55–61. 10.1097/00006205-199404000-00011 8035962

[B4] Butler R. N. (1963). The life review: an interpretation of reminiscence in the aged. Psychiatry 26, 65–76. 10.1080/00332747.1963.11023339 14017386

[B5] Cai M. (2018). Effects of individual reminiscence therapy on depression and loneliness in elderly patients with chronic diseases. Today Nurse. 25, 85–87.

[B6] Chen T. J. Li H. J. Li J. (2012). Research progress on the effect of reminiscence therapy on senile depression. Chinese J. Gerontol. 8, 1750–1754. 10.3969/j.issn.1005-9202.2012.08.108

[B7] Chiang K. J. Chu H. Chang H. J. Chung M. H. Chen C. H. Chiou H. Y.. (2010). The effects of reminiscence therapy on psychological well-being, depression, and loneliness among the institutionalized aged. Int. J. Geriatric Psychiatry 25, 380–388. 10.1002/gps.2350 19697299

[B8] Cotelli M. Manenti R. Zanetti O. (2012). Reminiscence therapy in dementia: a review. Maturitas 72, 203–205. 10.1016/j.maturitas.2012.04.008 22607813

[B9] Duru-Aşiret G. Kapucu S. (2016). The effect of reminiscence therapy on cognition, depression, and activities of daily living for patients with Alzheimer disease. J. Geriatr. Psychiatry Neurol. 29, 31–37. 10.1177/0891988715598233 26251112

[B10] Fan H. Y. Li Z. (2014). Advances in the use of reminiscence therapy in patients with dementia. Chinese J. Nurs. 49, 716–720. 10.3761/j.issn.0254-1769.2014.06.019

[B11] Fry P. S. (1983). Structured and unstructured reminiscence training and depression among the elderly. Clin. Gerontol. 1, 15–37. 10.1300/J018v01n03_06

[B12] Gil I. Costa P. Parola V. Cardoso D. Almeida M. Apóstolo J. (2019). Efficacy of reminiscence in cognition, depressive symptoms and quality of life in institutionalized elderly: a systematic review. Rev. Esc. Enferm. USP 53:e03458. 10.1590/s1980-220x2018007403458 30942298

[B13] Hamzehzadeh M. Golzari M. Rafiemanesh H. Meshki V. Abdolalizadeh M. Hoseini L.. (2018). Investigating the effectiveness of reminiscence therapy on of elderlies depression and optimism: an experiment study. Prensa Med. Argent. 104:6. 10.41720032/745X.1000318

[B14] Hang R. H. Liu X. M. Feng L. P. Xing J. (2011). Effects of psychological intervention on depressive symptoms, loneliness and well-being of empty nesters in the community. Chinese J. Gerontol. 31, 2723–2725. 10.3969/j.issn.1005-9202.2011.14.054

[B15] He F. T. (2018). A literature review of research on the effectiveness of reminiscence therapy in improving depression and depressive mood in older adults. Blooming Season 13:233. 10.3969/j.issn.1007-5070.2018.19.213

[B16] Higgins J. P. (2011). Cochrane Handbook for Systematic Reviews of Interventions. Version 5.1. 0 [Updated March 2011]. The Cochrane Collaboration. Available online at: http://www.cochrane-handbook.org (accessed May 12, 2021).

[B17] Higgins J. P. Altman D. G. Gøtzsche P. C. Jüni P. Moher D. Oxman A. D.. (2011). The Cochrane Collaboration's tool for assessing risk of bias in randomised trials. BMJ 343:d5928. 10.1136/bmj.d5928 22008217 PMC3196245

[B18] Housden S. (2009). The use of reminiscence in the prevention and treatment of depression in older people living in care homes: a literature review. Groupwork 19:28. 10.1921/095182410X490296

[B19] Hsieh H. F. Wang J. J. (2003). Effect of reminiscence therapy on depression in older adults: a systematic review. Int. J. Nurs. Stud. 40, 335–345. 10.1016/S0020-7489(02)00101-3 12667510

[B20] Kok R. M. Reynolds C. F. (2017). Management of depression in older adults: a review. JAMA 317, 2114–2122. 10.1001/jama.2017.5706 28535241

[B21] Lin Y. C. Dai Y. T. Hwang S. L. (2003). The effect of reminiscence on the elderly population: a systematic review. Public Health Nurs. 20, 297–306. 10.1046/j.1525-1446.2003.20407.x 12823790

[B22] McGirr A. Renaud J. Seguin M. Alda M. Benkelfat C. Lesage A.. (2007). An examination of DSM-IV depressive symptoms and risk for suicide completion in major depressive disorder: a psychological autopsy study. J. Affect. Disord. 97, 203–209. 10.1016/j.jad.2006.06.016 16854469

[B23] Onder G. Landi F. Gambassi G. Liperoti R. Soldato M. Catananti C.. (2005). Association between pain and depression among older adults in Europe: results from the Aged in Home Care (AdHOC) project: a cross-sectional study. J. Clin. Psychiatry 66, 982–988. 10.4088/JCP.v66n0804 16086612

[B24] Page M. J. McKenzie J. E. Bossuyt P. M. Boutron I. Hoffmann T. C. Mulrow C. D.. (2021). The PRISMA 2020 statement: an updated guideline for reporting systematic reviews. Syst. Rev. 10:89. 10.1186/s13643-021-01626-4 33781348 PMC8008539

[B25] Pot A. M. Bohlmeijer E. T. Onrust S. Melenhorst A. S. Veerbeek M. De Vries W. (2010). The impact of life review on depression in older adults: a randomized controlled trial. Int. Psychogeriatrics 22:572. 10.1017/S104161020999175X 20128949

[B26] Qin S. Liu S. Q. Bai H. Q. Yang J. H. (2017). Observation on effect of reminiscence therapy for reducing loneliness of the elderly in nursing institutions. Chinese Nurs. Res. 31, 590–591. 10.3969/j.issn.1009-6493.2017.05.024

[B27] Sahu I. Mohanty S. Pahantasingh S. (2019). Effect of reminiscence group therapy on depression, self-esteem and loneliness among elderly women residing in old age home. Int. J. Res. Med. Sci. 7:3685. 10.18203/2320-6012.ijrms20194293

[B28] Syed-Elias S. M. Neville C. Scott T. Petriwskyj A. (2020). The effectiveness of spiritual reminiscence therapy for older people with loneliness, anxiety and depression in Malaysia. J. Religion Spiritual. Aging 32, 341–356. 10.1080/15528030.2020.1765448

[B29] United Nations Department of Economic Social Affairs Population Division (2019a). World Population Prospects 2019: Highlights(ST/ESA/SER.A/423). Retrieved from: https://www.un.org/development/desa/publications/world-population-prospects-2019-highlights.html (accessed May12, 2021).

[B30] United Nations Department of Economic Social Affairs Population Division (2019b). World Population Ageing 2019: Highlights (ST/ESA/SER.A/430). Retrieved from: https://www.un.org/en/development/desa/population/publications/pdf/ageing/WorldPopulationAgeing2019-Highlights.pdf (accessed May12, 2021).

[B31] Viguer P. Satorres E. Fortuna F. B. Meléndez J. C. (2017). A follow-up study of a reminiscence intervention and its effects on depressed mood, life satisfaction, and well-being in the elderly. J. Psychol. 151, 789–803. 10.1080/00223980.2017.1393379 29166223

[B32] Wang L. L. Tan J. J. He C. L. Chen M. (2020). Effects of reminiscence therapy on depression and life satisfaction in elderly patients with depression. Nurs. Rehabil. J. 19, 65–67. 10.3969/j.issn.1671-9875.2020.09.018

[B33] Wang W. Wang H. C. Chen Z. D. (2007). Phylogeny and morphological evolution of tribe Menispermeae (Menispermaceae) inferred from chloroplast and nuclear sequences. Perspect. Plant Ecol. Evol. Syst. 8, 141–154. 10.1016/j.ppees.2006.12.001

[B34] Wang X. Zhang J. F. Li Z. J. Wang F. Y. (2011). Influence of reminiscence therapy on depression and happiness degree of empty nest elderly in community. Chinese Nurs. Res. 25, 3192–3194. 10.3969/j.issn.1009-6493.2011.34.040

[B35] Wang X. R. Zhang W. H. (2019). Current situation and related factors of elderly depression in rural areas. Smart Healthcare 5, 33–35. 10.19335/j.cnki.2096-1219.2019.28.015

[B36] Watt L. M. Cappeliez P. (2000). Integrative and instrumental reminiscence therapies for depression in older adults: intervention strategies and treatment effectiveness. Aging Mental Health, 4, 166–177. 10.1080/13607860050008691

[B37] Westerhof G. J. Bohlmeijer E. Webster J. D. (2010). Reminiscence and mental health: a review of recent progress in theory, research and interventions. Ageing Soc. 30:697. 10.1017/S0144686X09990328

[B38] World Health Organization (2014). Preventing Suicide: A Global Imperative. World Health Organization. Retrieved from: https://apps.who.int/iris/handle/10665/131056; (accessed May 12, 2021).

[B39] Wu D. M. Hu X. Y. (2017). Current status and prospects of the application of reminiscence therapy in China. Chinese J. Gerontol. 37, 3893–3895. 10.3969/j.issn.1005-9202.2017.15.111

[B40] Yue Y. Chen Y. Y. (2013). Effect of structured group reminiscence on depressive symptoms and life satisfaction in elders. China Modern Doctor 51, 110–112.

[B41] Zhou W. He G. Gao J. Yuan Q. Feng H. Zhang C. K. (2012). The effects of group reminiscence therapy on depression, self-esteem, and affect balance of Chinese community-dwelling elderly. Arch. Gerontol. Geriatr. 54, 440–447. 10.1016/j.archger.2011.12.003 22206591

